# Study on Hypervelocity Impact Characteristics of Polypropylene Spheres on Whipple Shields

**DOI:** 10.3390/polym17030319

**Published:** 2025-01-24

**Authors:** Yuxiang Zhang, Shengjie Wang, Runqiang Chi, Qingxu Liu, Xiaodan Li, Chunlei Kan, Sainan Xu, Jun Ma

**Affiliations:** 1 Beijing Machine and Equipment Institute, Beijing 100854, China; naruto114@163.com (Y.Z.); youaresmart@126.com (S.W.); liuqingxubuaa@foxmail.com (Q.L.); lixd1112@163.com (X.L.); kchunlei@163.com (C.K.); xusnbit@163.com (S.X.); chinamajun@163.com (J.M.); 2 Hypervelocity Impact Research Center, Harbin Institute of Technology, Harbin 150080, China

**Keywords:** hypervelocity impact, polypropylene sphere, damage characteristics, Whipple shield

## Abstract

The hypervelocity impact characteristics of 7 mm diameter polypropylene spheres on Whipple shields with different bumper thicknesses at a velocity ranging from 2.257 to 4.012 km/s are studied in this work. The study has found that the hypervelocity impact characteristics of the polypropylene sphere contrast markedly with those of the aluminum sphere. The polypropylene sphere transfers greater energy to the bumper upon impact, leading to a larger perforation. Meanwhile, the microstructure at the perforation site is complex. The polypropylene sphere undergoes a phase change after impacting the bumper, forming a debris cloud primarily composed of ‘liquid filaments’. This debris cloud creates a ‘groove’-shaped damage morphology on the rear wall. The polypropylene sphere exhibits weaker perforation capabilities but generates a more extensive damage area on the rear wall.

## 1. Introduction

In recent years, the aerospace industry across various nations has experienced rapid development, with a succession of advanced spacecraft emerging, significantly enhancing human life and work. However, this progress inevitably contributes to the continuous degradation of the space environment [1]. Polymers, due to their exceptional physical and chemical properties, have increasingly supplanted conventional metals in numerous spacecraft systems, including satellites, rockets, and spaceships [2,3,4]. When polymer-based components in spacecraft encounter collisions or other disintegration causes, substantial amounts of polymer space debris are generated. Existing research on space debris impacts and shield structures predominantly focuses on metal debris [5], with little attention given to polymer debris. Consequently, current shield structures may not be adequately equipped to address the increasingly complex space debris environment of today and the future.

To study the behavior of polymer materials under ultra-high strain rate conditions, a series of nanoscale impact experiments have been meticulously conducted by researchers [6,7,8,9]. These studies collectively yielded valuable insights into material responses at exceptionally high strain rates, reaching up to 10^7^–10^8^ s^−1^. Due to the heightened sensitivity of polymers to strain rate, under hypervelocity impact, polymer target plates exhibit varying damage forms at different distances from the impact site [10,11,12]. Dewapriya [13] further studied the energy dissipation effect of polyurea layers at the nanoscale. Although nanoscale tests cannot represent the macroscopic properties of materials, they can, to a certain extent, reveal the response patterns of materials under extremely high strain rates.

In space debris protection research, numerous scholars assert that polymer materials, owing to their exceptional strength, toughness, and low density, can potentially replace existing space debris shield structures. Cha [14] conducted hypervelocity impact tests at different temperatures, showing that ultra-high molecular weight polyethylene has better impact resistance than Kevlar at the same surface density. Kawai [15] employed two imaging techniques to document the shock wave propagation and damage evolution processes in a polycarbonate target subjected to impacts by an alumina ceramic sphere at different velocities. Callahan [16] further conducted research on the response characteristics of polycarbonate under impact velocities ranging from 0.4 to 6.5 km/s. Rogers et al. [17,18] revealed that variations in polymer molecular structure significantly influence their macroscopic performance through a comparative analysis of hypervelocity impacts on two varieties of polyethylene shield structures. Bowering [19] researched the energy dissipation capabilities of poly(methyl methacrylate) under impact velocities ranging from 2 to 7 km/s.

Some researchers have conducted studies on the hypervelocity impact characteristics of polymer debris. Drawing from experiments and numerical simulation results, Li et al. [20,21] derived the ballistic limit curve for the hypervelocity impact of the polypropylene sphere on the Whipple shield, demonstrating that, under specific conditions, the damage ability of the polypropylene sphere is inferior to that of aluminum. Song [22] conducted hypervelocity impact experiments using a mylar flyer plate on a Whipple shield, obtaining the ballistic limit curve within the 3–12 km/s impact velocity range. Shojaei [23] explored the cratering features of polycarbonate projectiles when they undergo hypervelocity impact with thick plates. Akahoshi [24] examined the distribution patterns of fragments that result from the impact and subsequent fragmentation of polyethylene projectiles.

Current research has made significant progress in understanding the response and protective characteristics of polymers under ultra-high strain rates when used as target plates. However, there is a notable dearth of research in which polymers serve as the active impacting agents, and systematic, regular research outcomes in this domain are conspicuously absent. This work investigates the hypervelocity impact of a polypropylene sphere on a Whipple shield, detailing the damage and debris cloud characteristics under various impact conditions. The results are then compared in detail with those from the impacts of aluminum spheres under the same conditions. The insights garnered from this research can enhance the understanding of polymer behavior under ultra-high strain rates and offer valuable guidance for improving the protective efficacy of existing spacecraft shielding structures.

## 2. Materials and Methods

### 2.1. Experimental Setup

**Figure 1:**
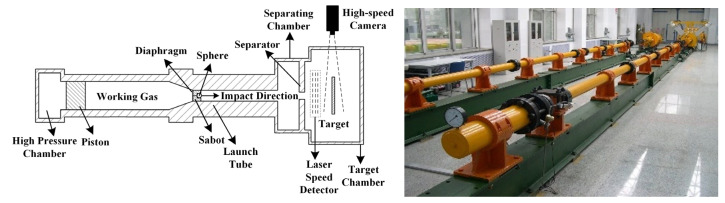
Schematic diagram and equipment of hypervelocity impact experiment.

The hypervelocity impact experiments described in this work were conducted using the two-stage light gas gun at the Hypervelocity Impact Research Center, Harbin Institute of Technology. The experimental equipment and principles are illustrated in Figure 1. The first-stage high-pressure gas is nitrogen, with a pressure range of 4–8.5 MPa in the study. The second-stage working gas is hydrogen, with a pressure of 0.1 MPa before compression. Initially, high-pressure gas is introduced into the high-pressure chamber to propel the piston within the tube, rapidly compressing the working gas. Upon reaching a specific pressure threshold, the diaphragm ruptures. Consequently, the high-pressure working gas enters the launch tube, propelling the sabot and sphere into the separating chamber. In this chamber, aerodynamic forces separate the sabot from the sphere. The sphere then traverses a laser speed detector and proceeds to the target chamber, impacting the target plate. A high-speed camera positioned perpendicular to the impact direction captures the sequence at 600,000 frames per second.

**Figure 2:**
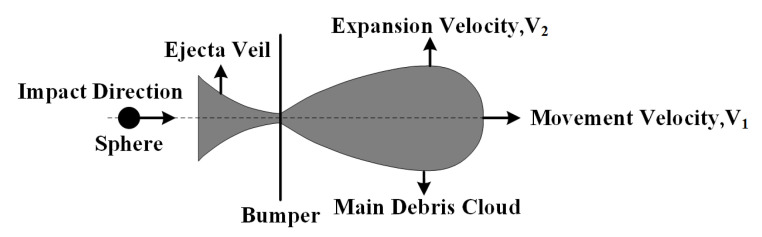
Schematic diagram of the debris cloud velocity.

Following the experiment, the diameter of the bumper perforation $D_{b}$ was measured with a digital microscope. The extent of damage to the rear wall was assessed using both a digital microscope and a confocal laser scanning microscope. The micromorphology of the damage was evaluated via a scanning electron microscope. The velocity of the debris cloud was determined by analyzing images captured by a high-speed camera. The movement velocity $V_{1}$ refers to the movement velocity of the front endpoint of the debris cloud in the direction of the impact, while the expansion velocity $V_{2}$ denotes the velocity of the upper endpoint of the debris cloud in the direction perpendicular to the impact, as illustrated in Figure 2.

### 2.2. Status of the Polypropylene Sphere and the Whipple Shield

**Figure 3:**
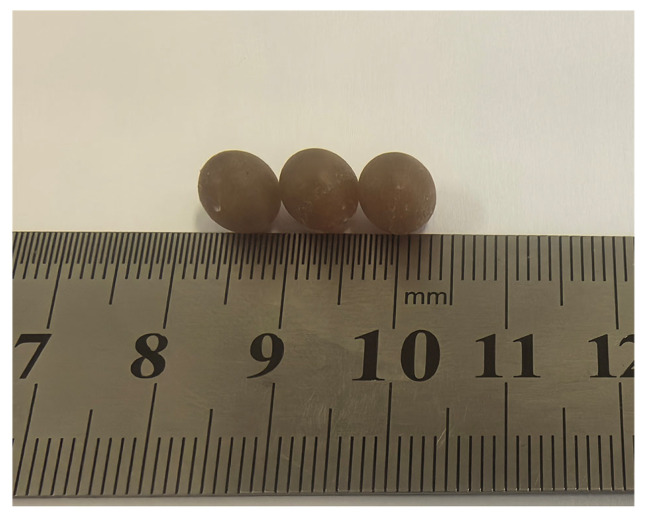
Polypropylene spheres image.

**Figure 4:**
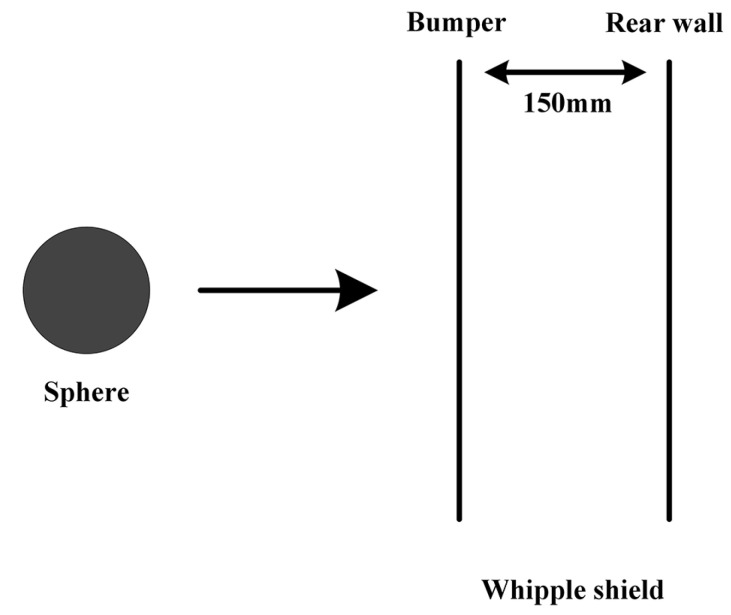
Schematic diagram of the Whipple shield.

The polypropylene sphere shown in Figure 3 is produced via injection molding. The diameter of the sphere D is 7 mm. The target is a Whipple shield structure composed of Al 5A06, which is manufactured by CHINALCO in Chongqing, China, measuring 200 mm × 200 mm with a spacing of 150 mm, as shown in Figure 4. Four hypervelocity impact experiments were conducted, and the parameters of the sphere and target are presented in Table 1.

## 3. Results

This section offers a comprehensive introduction to the characteristics of the bumper damage, the debris cloud, and the rear wall damage caused by the polypropylene sphere impacting the Whipple shield. The findings are compared with those involving the aluminum sphere under identical impact conditions. This work selects two comparison criteria: identical mass and identical volume. Under the same mass, corresponding to equivalent kinetic energy, the emphasis is on comparing the effects of debris material on fragmentation, phase transition extent, and penetration capacity of the Whipple structure. In the same volume condition, the focus shifts to comparing the effects of debris material on debris cloud velocity and the extent of the damage area on the Whipple shield.

### 3.1. Bumper Damage Characteristics

**Figure 5:**
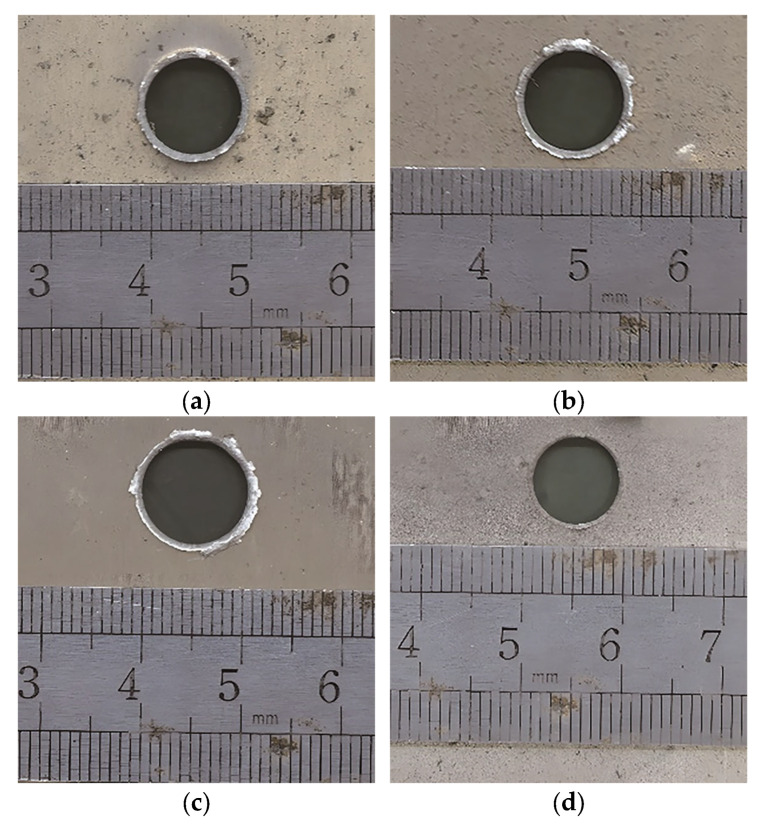
Bumper perforation images for Test 1 to Test 4. (**a**) Test 1; (**b**) Test 2; (**c**) Test 3; (**d**) Test 4.

Figure 5 presents the bumper perforation images from Test 1 to Test 4. The perforation caused by the impact of a polypropylene sphere is circular. Influenced by dynamic shear failure and inertia effects, the perforation diameter exceeds the sphere’s, demonstrating a significant hole-expanding effect [25,26]. As shown in Figure 5a–c, when the bumper thickness, t, is 1 mm, the perforation edges display prominent overturned flaps and material detachment. Conversely, as shown in Figure 5d, when the bumper thickness is 0.5 mm, the perforation edges are clean, with no overturned flap phenomenon. Upon the hypervelocity impact of the sphere onto the bumper, the bumper undergoes substantial plastic deformation and generates an intense shock wave within itself. This shock wave radiates outward from the area of impact, exerting significant, outwardly directed radial forces on the material surrounding the interior of the penetration hole. Consequently, the material at the periphery of the penetration hole is subjected to compression and deformation, resulting in its overturning and spalling [27]. The hole-expanding effect and overturned flap phenomenon align with those observed in bumper perforations caused by the impact of an aluminum sphere [28].

**Figure 6:**
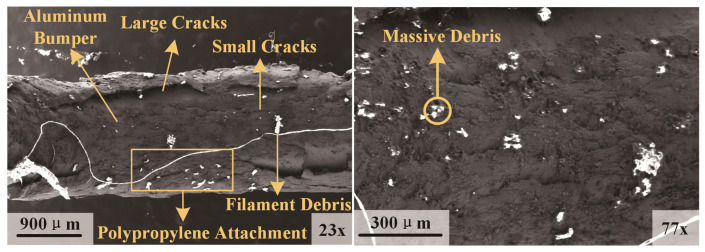
Micromorphology of the bumper perforation in Test 2.

Figure 6 shows the micromorphology of the bumper perforation in Test 2. The hole wall surface is coated with substantial amounts of ‘filamentous’ and ‘massive’ polypropylene debris. The wall surface, affected by the impact, exhibits numerous cracks, with larger cracks at the overturned flaps on both sides. The intersection of these multidirectional cracks can lead to material detachment. Regarding micromorphology, the perforation walls caused by the aluminum sphere remain smooth with minimal adherence of spherical debris. Conversely, the polypropylene sphere leads to a more intricate micromorphology of the bumper perforation.

Table 2 presents the diameter of bumper perforation and the normalized diameter of bumper perforation from Test 1 to Test 4. The experimental results indicate that the perforation diameter enlarges with a constant bumper thickness as impact velocity $V_{0}$ rises. Conversely, when impact velocities are comparable, the perforation diameter increases with increasing bumper thickness.

**Figure 7:**
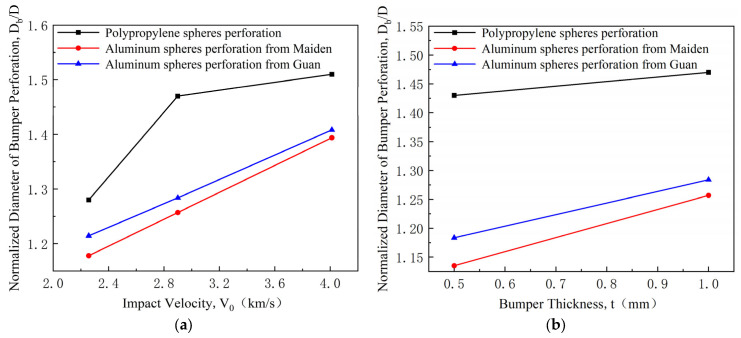
Normalized diameter of bumper perforation by polypropylene and aluminum spheres under the same volume conditions. (**a**) Normalized diameter of bumper perforation at various impact velocities; (**b**) Normalized diameter of bumper perforation at various bumper thicknesses.

The density of the polypropylene sphere in this work is 0.874 g/cm^3^, while aluminum has a density of 2.64 g/cm^3^. The polypropylene sphere with a diameter of 7 mm corresponds to an aluminum sphere of the same mass with a diameter of approximately 4.84 mm. According to existing results [27], under identical mass conditions, the perforation size caused by the polypropylene sphere on the bumper is significantly larger than that caused by the aluminum sphere due to the difference in sphere size. To analyze the impact of sphere material on the perforation size of the bumper, this work primarily compares the perforation sizes caused by the two types of spheres under identical volume conditions, as illustrated in Figure 7. The perforation data for the aluminum sphere are derived from Maiden’s model [29]:

(1) $$\frac{D_{b}}{D}=2.4\frac{V_{0}}{C}{\left(\frac{t}{D}\right)}^{2/3}+0.9$$

and the Guan’s model [28]:

(2) $$\frac{D_{b}}{D}=2.19{\left(\frac{V_{0}}{C}\right)}^{1.12}{\left(\frac{t}{D}\right)}^{0.7}+1$$

where D, $D_{b}$, $V_{0}$, t and C, respectively, represent the diameter of the sphere, the diameter of the bumper perforation, the impact velocity, the bumper thickness, and the sound speed of the bumper material, with aluminum’s sound speed specified as 5.328 km/s [28].

As illustrated in Figure 7, under identical volume conditions, the normalized diameter of the bumper perforation of the polypropylene sphere curve consistently exceeds that of the aluminum sphere, indicating a relatively larger perforation diameter caused by the polypropylene sphere. Consequently, the bumper damage inflicted by the impact of the polypropylene sphere is more severe than that caused by the aluminum sphere.

### 3.2. Debris Cloud Characteristics

**Figure 8:**
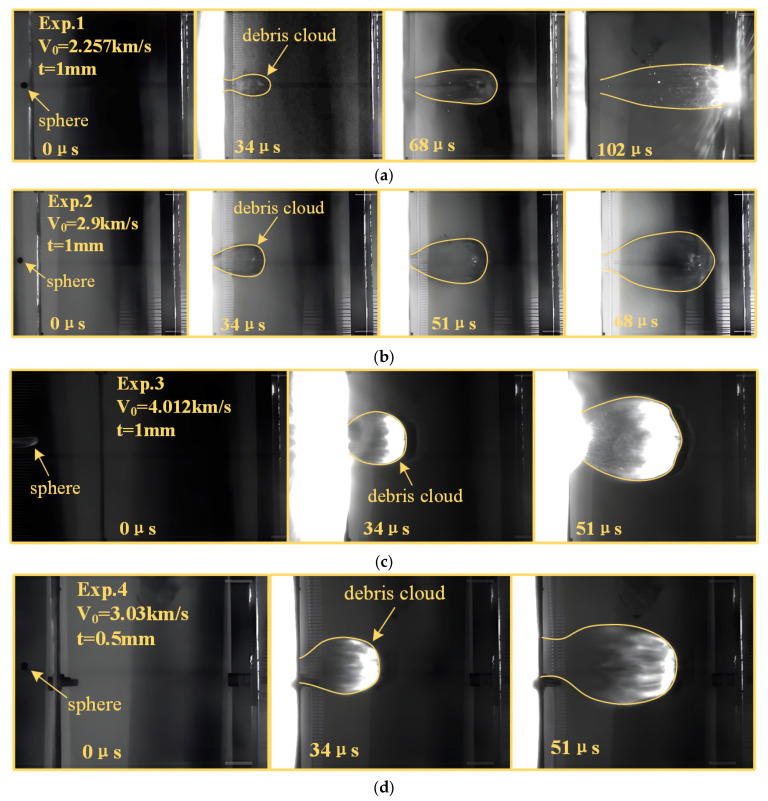
Debris cloud images from Test 1 to Test 4. (**a**) Test 1; (**b**) Test 2; (**c**) Test 3; (**d**) Test 4.

The debris cloud images from Test 1 to Test 4 are shown in Figure 8. The images reveal that the sphere remains intact before impacting the bumper. At an impact velocity of 4.012 km/s, the sphere produces a flash accompanied by a trail, caused by friction-induced heating as it traverses the separating chamber at high velocity. Upon impacting the bumper, a significant flash occurs on the front side, and a ‘balloon’-shaped debris cloud appears on the rear. The debris cloud expands uniformly between the bumper and rear wall until it impacts the rear wall. Figure 8b,c show that the front end of the debris cloud is darker or has a higher flash intensity, indicating that the debris is concentrated with mass in this region. As shown in Figure 8a, it is evident that after impacting the bumper, the polypropylene undergoes a phase change, forming ‘liquid filamentous’ debris, which has a morphology similar to the ‘Fluid like’ fragments in the Callahan’s [16] tests.

The formation of ‘liquid filamentous’ debris is due to the increase in internal energy of the sphere during impact, causing polypropylene to change from a solid state to a viscous state. This change occurs owing to the unique intermolecular forces of polypropylene, which impart significant viscoelasticity and orientation to its viscous state. Consequently, debris in this state exhibits exceptionally high elongational viscosity in a specific direction, inhibiting the liquid from fracturing along that axis. Thus, unlike conventional Newtonian liquids that readily break into droplets under external forces, polypropylene liquid forms distinctive ‘liquid filament’ debris due to the influence of robust orientation and elongational viscosity [30,31,32].

By comparing experimental images under varying impact conditions, it is evident that as impact velocity and bumper thickness increase, the radial expansion of the debris cloud becomes more pronounced, and the damage area on the rear wall progressively enlarges. This phenomenon primarily arises from the greater allocation of increased shock wave intensity to the debris cloud’s expansion velocity [33].

Table 3 presents the debris cloud velocity from Test 1 to Test 4. The experimental results indicate that, with a constant bumper thickness, the debris cloud’s movement velocity and expansion velocity increase approximately linearly with impact velocity. Conversely, when impact velocities are comparable, the debris cloud’s movement and expansion velocity decrease as bumper thickness increases.

**Figure 9:**
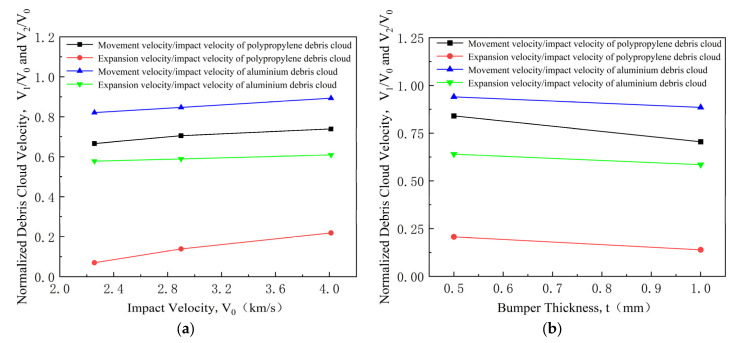
Normalized debris cloud velocity of polypropylene and aluminum spheres. (**a**) Normalized debris cloud velocity at various impact velocities; (**b**) Normalized debris cloud velocity at various bumper thicknesses.

The velocity of the debris cloud is significantly affected by the ratio of the bumper’s thickness to the sphere’s diameter. Therefore, this work mainly compares the debris cloud velocities of polypropylene and aluminum spheres under identical volume conditions. The debris cloud velocity for the aluminum sphere is extrapolated from Chi’s experimental data [34]. As shown in Figure 9, under identical impact conditions, the movement and expansion velocities of the debris cloud from the polypropylene sphere are lower than those from the aluminum sphere.

### 3.3. Rear Wall Damage Characteristics

**Figure 10:**
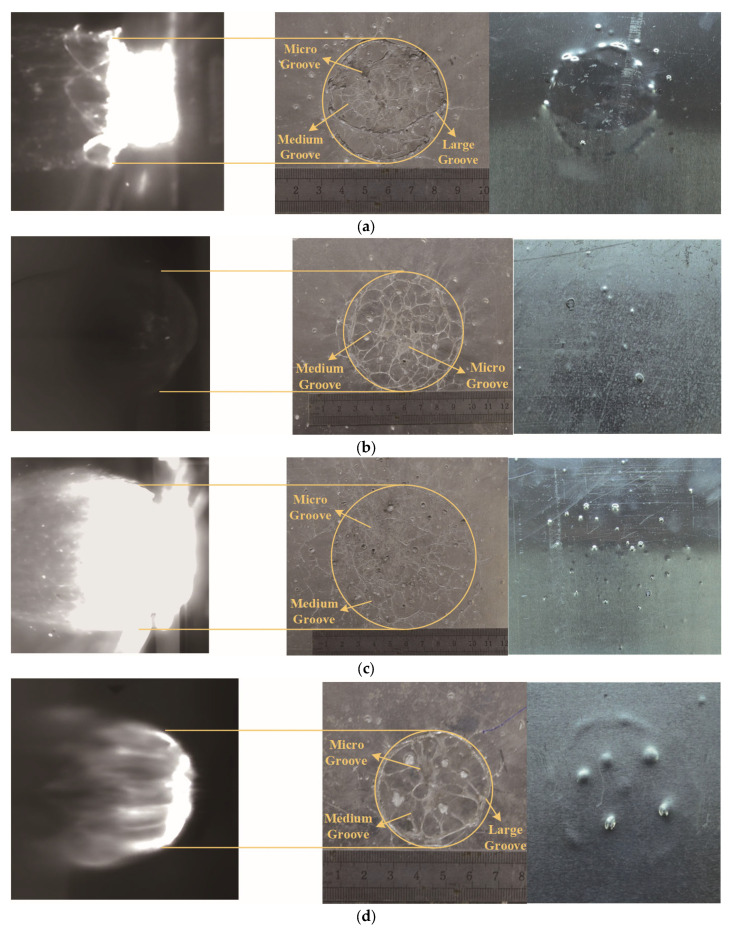
Rear wall damage morphology and corresponding location of debris cloud from Test 1 to Test 4. (**a**) Test 1; (**b**) Test 2; (**c**) Test 3; (**d**) Test 4.

Figure 10 illustrates the morphology of rear wall damage from Test 1 to Test 4, alongside the corresponding positions of the debris cloud. The figure reveals that the damage is predominantly concentrated in a circular area at the center of the rear wall. The damage morphology encompasses ‘grooves’ formed by the impact of polypropylene ‘liquid filaments’, craters resulting from the impact of bumper debris, and a combination thereof. The correlation between the damage area and the debris cloud’s position indicates that significant damage to the rear wall predominantly occurs in regions with a higher concentration of fragments and mass at the front end of the debris cloud. The ‘grooves’ can be categorized into three types based on width. Large ‘grooves’, ranging from 1 to 2 mm in width and 0.2–1 mm in depth, are arranged in a circular or arc pattern outside the damage area. Medium ‘grooves’, spanning 0.1–1 mm in width and 0.1–0.6 mm in depth, exhibit a ‘spider web’ pattern throughout the entire damage area. Micro ‘grooves’, irregularly and densely distributed at impact velocities between 2.257 and 3.03 km/s, become scattered near some medium ‘grooves’ when the impact velocity reaches 4.012 km/s, with width and depth less than 0.1 mm. The backside of the rear wall displays pronounced annular, granular bulges or spalling, corresponding to the large ‘grooves’ and deeper craters on the front side.

**Figure 11:**
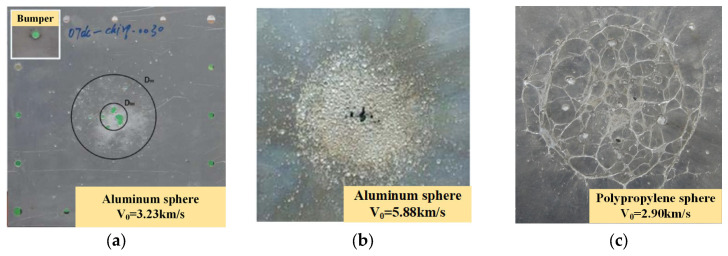
The damage morphology of polypropylene and aluminum spheres impacts the rear wall at different velocities. (**a**) The damage morphology of the rear wall was formed by the impact of an aluminum sphere at a velocity of 3.23 km/s [35]; (**b**) The damage morphology of the rear wall was formed by the impact of an aluminum sphere at a velocity of 5.88 km/s [28]; (**c**) The damage morphology of the rear wall for Test 2.

Figure 11 illustrates the damage morphology on the rear wall caused by impacts from aluminum and polypropylene spheres. Figure 11a demonstrates the damage morphology of the rear wall caused by an aluminum sphere impacting the Whipple shield at a velocity of 3.23 km/s. Upon impact with the bumper, the aluminum sphere shatters without undergoing a phase change, forming a solid debris cloud that results in craters or perforations on the rear wall. Figure 11b illustrates the damage morphology of the rear wall caused by an aluminum sphere impacting the Whipple shield at a velocity of 5.88 km/s. In this scenario, the aluminum sphere undergoes a phase change upon impact with the bumper, transforming into liquid droplets. These droplets then create craters or spalling on the rear wall. Figure 11c illustrates the damage morphology of the rear wall resulting from the impact of a polypropylene sphere in Test 2. Upon striking the bumper, the polypropylene sphere undergoes a phase transition, generating ‘liquid filament’ debris, leading to distinctive ‘grooves’-shaped damage on the rear wall. Compared to the polypropylene sphere, the damage caused by the aluminum sphere is more concentrated at the impact center and less so at the edges, while the damage from the polypropylene sphere is less intense at the center and more pronounced at the edges. Owing to the relatively low melting point of polypropylene, the temperature rise induced at an impact velocity of 2.9 km/s is sufficient to reach the material’s phase transition temperature. Consequently, upon impact, the polypropylene transitions from a solid to a fluid state. The fluid polypropylene, characterized by its higher viscosity and lower surface tension compared to liquid aluminum, is less likely to form droplets. Instead, it tends to form filaments during the expansion and propagation of the debris cloud. When these filaments strike the rear wall, they create ‘grooves’. In contrast, the debris cloud generated by the aluminum sphere—whether consisting of solid fragments at lower impact velocities or liquid droplet fragments resulting from phase transition at higher velocities—typically results in the formation of craters or penetration holes when they impact the rear wall.

**Figure 12:**
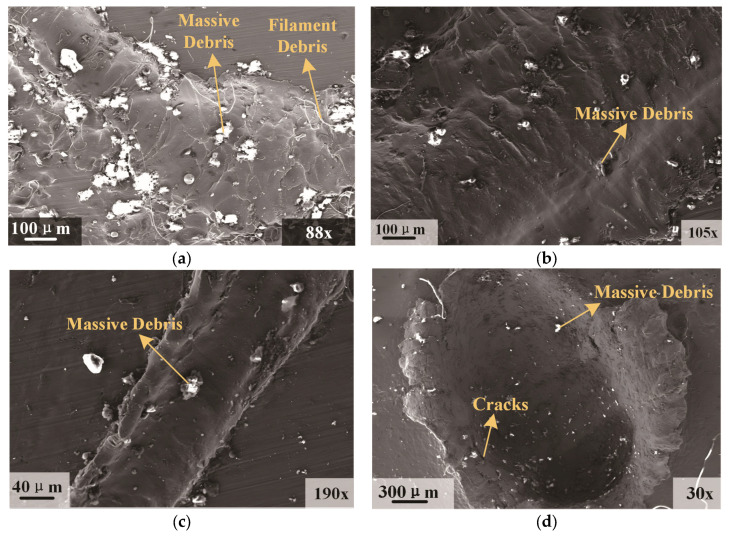
Micromorphology of the rear wall damage area. (**a**) Micromorphology of the large ‘grooves’; (**b**) Micromorphology of the medium ‘grooves’; (**c**) Micromorphology of the micro ‘grooves’; (**d**) Micromorphology of the crater.

Figure 12 illustrates the micromorphology of the damage area on the rear wall. Figure 12a–c, respectively, showed the micromorphology of varying sizes of ‘grooves’. The larger the ‘groove’, the more intricate its internal morphology becomes. Large ‘grooves’ encompass substantial ‘filamentous’ and ‘massive’ debris, with rough surfaces at both the bottom and side walls, clearly exhibiting damage morphology induced by liquid impact [36]. Medium ‘grooves’ contain relatively less debris, featuring smoother bottoms and less rough side walls. Micro ‘grooves’ further minimize debris, presenting smooth surfaces at the bottom and side walls. Figure 12d reveals the micromorphology of a crater, characterized by a small amount of the ‘massive’ debris, smooth surfaces, and a few cracks near the side walls and edges. Compared to craters caused by an aluminum sphere, the micromorphology within the rear wall damage area induced by the polypropylene sphere is relatively more complex due to the intricate morphology of debris post-phase change. The formation of ‘grooves’ is predominantly influenced by the kinetic energy of the impacting filaments. Specifically, filaments with larger dimensions and higher kinetic energy tend to produce ‘grooves’ of greater size. Moreover, the substantial impact force can induce additional deformation and the formation of cracks in the material comprising the ‘groove’ walls and bottom. Polypropylene fluid, which possesses a degree of viscosity, causes some fragments to adhere to the ‘grooves’ post-impact. This adhesion contributes to a more intricate microstructure within the ‘grooves’.

**Figure 13:**
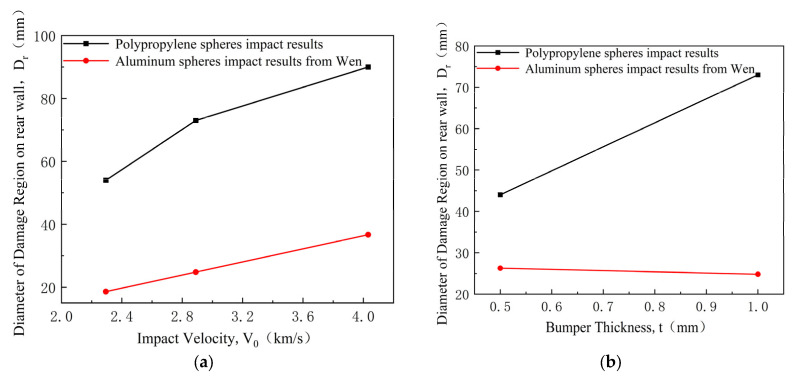
Diameter of the rear wall damage area of polypropylene and aluminum spheres. (**a**) Diameter of the rear wall damage area at various impact velocities; (**b**) Diameter of the rear wall damage area at various bumper thicknesses.

Figure 13 compares the diameter of the rear wall damage area $D_{r}$ caused by polypropylene and aluminum spheres. The rear wall damage area caused by the aluminum sphere is calculated using Wen’s model [35]:

(3) $$\frac{D_{r}-D}{S}=0.37\frac{V_{0}}{C}-0.082$$

where S denotes the distance between the bumper and the rear wall.

It can be observed that the diameter of the rear wall damage area caused by the polypropylene sphere increases with the impact velocity and bumper thickness. Compared to the aluminum sphere under the same impact conditions, the diameter of the rear wall damage area caused by the polypropylene sphere is relatively larger. Moreover, changes in bumper thickness have a more significant impact on the diameter of the rear wall damage area caused by the polypropylene sphere than the aluminum sphere.

Table 4 illustrates the maximum average ‘groove’ depth from Test 1 to Test 4. This metric is derived by averaging the depths of the five deepest ‘grooves’ on the rear wall surface. With a consistent bumper thickness, the maximum average ‘groove’ depth diminishes as impact velocity increases. Conversely, when impact velocities are comparable, this depth decreases with greater bumper thickness. According to Reimerdes’s limit impact equation [37], under identical impact conditions, a 4.84 mm diameter aluminum sphere can perforate the rear plate, whereas the ‘groove’ depth caused by the polypropylene sphere is substantially less than the thickness of the rear plate. The perforation capability of the Whipple shield by the polypropylene sphere markedly differs from that of the aluminum sphere.

## 4. Discussion

### 4.1. Analysis Based on the Shock Wave Theory

**Figure 14:**
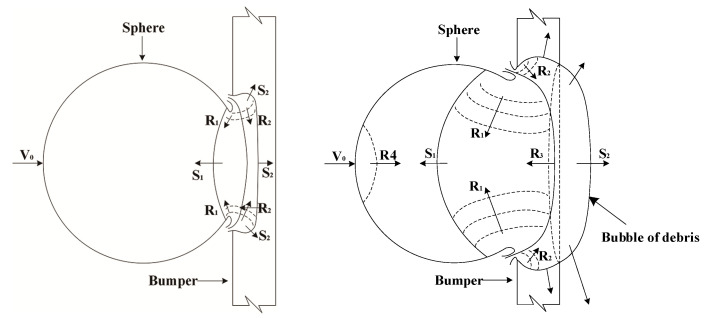
Schematic diagram of shock wave propagation in the hypervelocity impact.

The damage characteristics on the Whipple shield caused by the impact of a polypropylene sphere can be qualitatively analyzed and illustrated through the principles of shock wave propagation [38,39]. As illustrated in Figure 14, when a sphere impacts a thin plate, two shock waves, $S_{1}$ and $S_{2}$, are generated at the contact interface between the sphere and the plate, propagating in opposite directions. Under the influence of these shock waves, the sphere and the plate materials undergo significant compression, accompanied by shear failure. Upon reaching the free surface, the shock wave reflects and forms the rarefaction wave [40]. $R_{1}$ to $R_{4}$ denote the rarefaction waves reflected from the sphere’s surface, the front surface of the bumper, the rear surface of the bumper, and the rear surface of the sphere, respectively. The intensity of these rarefaction waves is directly correlated with the intensity of the initial shock waves [41]. A rarefaction wave is a tensile wave. The interaction of rarefaction waves originating from different surfaces can generate substantial tensile forces. When these tensile forces surpass the material’s yield strength, they can cause tensile failure. During propagation, the rarefaction wave will overtake and unload the shock wave [42].

**Figure 15:**
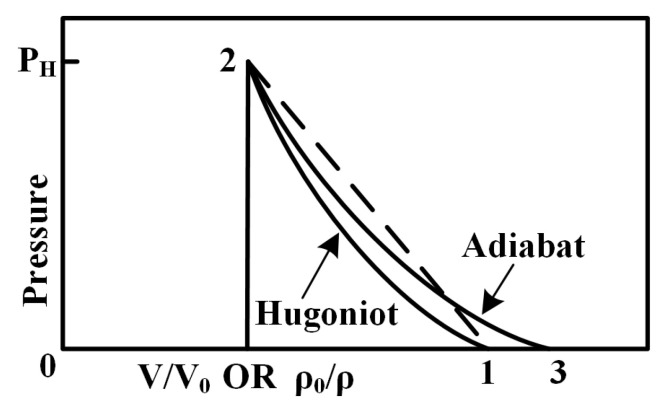
Pressure and volume variation curves during shock wave propagation [38].

As shown in Figure 15, the loading process of the shock wave in the sphere and the bumper can be approximately regarded as adiabatic and non-isentropic, represented by the area enclosed by dashed line 1–2. The unloading process of the rarefaction wave, in contrast, can be considered adiabatic and isentropic, delineated by the area within curve 2–3. The difference between these areas signifies the increase in the material’s internal energy resulting from the shock wave’s loading and unloading processes. This energy increase raises the material’s temperature, leading to phase transitions once a critical temperature threshold is reached [43]. The propagation and interaction of the shock wave within the sphere and the bumper dictate the size of the fragments and the extent of phase transitions induced by the impact [44].

Under the assumptions of fluid dynamics, the basic relationship of shock waves in solids and the relationship between shock wave velocity and particle velocity are as follows [45]:

(4) $$\rho_{0}(U-u_{0})=\rho (U-u)$$

(5) $$p-p_{0}=\rho_{0}(U-u_{0})(u-u_{0})$$

(6) $$e+\frac{p}{\rho }+\frac{1}{2}{\left(U-u\right)}^{2}=e_{0}+\frac{p_{0}}{\rho_{0}}+\frac{1}{2}{\left(U-u_{0}\right)}^{2}$$

(7) U=C+su

(8) $$V_{0}=u_{P}+u_{T}$$

where ρ, u, p and e, respectively, represent the density, particle velocity, pressure, and internal energy of the material. The subscript 0 denotes the state of the material before the wave front, while the absence of a subscript indicates the state after the wave front. The subscripts P and T correspond to the parameters of the sphere and the bumper. U represents the shock wave velocity, and C, S are material parameters. The shock temperature and residual temperature of the material need to be calculated by combining thermodynamic equations or equations of state with the Hugoniot curve. Based on the shock compression line p(v) and empirical relationships $\gamma /v=\gamma_{0}/v_{0}$, the shock temperature can be derived as follows:

(9) $$p(v)=\frac{C^{2}(v_{0}-v)}{{\left[v_{0}-s(v_{0}-v)\right]}^{2}}$$

(10) $$T=T_{0}\exp(\gamma_{0}\eta )+\frac{C^{2}}{C_{v}}\exp(\gamma_{0}\eta )\int_{0}^{\eta }\frac{sx^{2}}{{\left(1-sx\right)}^{3}}\exp(-\gamma_{0}x)dx$$

(11) $$\eta =1-\frac{v}{v_{0}}=1-\frac{\rho_{0}}{\rho }$$

where γ, v, T and $C_{v}$, respectively, represent the Gruneisen coefficient, specific volume, temperature, and specific heat. As entropy increases during compression, the material, upon unloading from the shock-compressed state along the isentropic path to a zero-pressure state, fails to restore its specific internal energy to the initial level. Consequently, the unloading specific volume and temperature surpass their initial values. The discrepancy between the unloading temperature and the initial temperature is referred to as the residual temperature. The isentropic unloading line is mathematically expressed as:

(12) $$v_{r}=v_{0}\left\{1+\alpha \left[T\exp(\frac{\gamma_{0}}{v_{0}}(v-v_{0}))-T_{0}\right]\right\}$$

(13) $$T_{r}=T\exp\left[\frac{\gamma_{0}}{v_{0}}(v-v_{r})\right]$$

The subscript r represents the state after isentropic unloading, and α is the coefficient of thermal expansion of the material.

To simplify the calculation, the material parameters ignore variations with temperature and pressure, as well as the latent heat of phase change. The material parameters of polypropylene and aluminum are detailed in Table 5 [20,46,47,48,49]. Based on these parameters, the state of the polypropylene under various impact conditions can be determined. The specific results are shown in Table 6.

According to the effective thickness theory, when the bumper is relatively thin, the rarefaction wave generated at the free surface of the bumper overtakes the shock wave within the sphere before it reaches the free surface. In this scenario, as the thickness of the bumper increases, the distance required for the rarefaction wave to overtake the shock wave also increases. Consequently, the shock wave travels farther within the sphere, leading to more extensive fragmentation and phase transitions. Conversely, when the bumper thickness is relatively thick, the rarefaction wave fails to overtake the shock wave before it reaches the free surface. In such cases, further increases in the bumper thickness have negligible influence on the shock wave’s behavior within the sphere. Maiden and McMillan [29] examined the rarefaction wave speed, and Wen [42] has developed calculation models to determine its interaction with shock waves:

(14) $$c_{as}=U\text{×}{\left[0.49+{\left(\frac{U-u}{U}\right)}^{2}\right]}^{1/2}$$

(15) $$\frac{x_{M}}{D}=\frac{U_{P}c_{asP}(U_{T}-u_{T}+c_{asT})t}{U_{T}c_{asT}(U_{P}-u_{P}+c_{asP})D}$$

where $c_{as}$ and $x_{M}$, respectively, represent the rarefaction wave speed and the distance from the initial impact point to the location where the axial rarefaction wave overtakes the shock wave. The location of the intersection under varying impact conditions is determined using Equations (14) and (15), as summarized in Table 7.

The results indicate that an increasing impact velocity leads to higher shock wave pressure generated by the sphere–target impact, resulting in larger bumper perforation, faster debris cloud velocity, and a larger damage area on the rear wall. Simultaneously, both the shock temperature and the residual temperature rise. At an impact velocity of 2.257 km/s, the shock temperature reaches 584.2 K, and the residual temperature is 452.6 K, both surpassing the melting point of polypropylene. This suggests that polypropylene undergoes a phase transition under these conditions, forming a ‘liquid filament’ like fluid debris cloud due to its unique intermolecular forces. As impact velocity increases, the sphere’s fragmentation and phase transition become more pronounced, producing smaller sphere fragments and more fluid debris. Consequently, the ‘groove’ type damage on the rear wall diminishes in size.

As shown in Table 7, across the four tests, the distance between the point where the rarefaction wave overtakes the shock wave and the initial impact point is smaller than the sphere’s diameter. This reveals that the shock wave generated within the sphere is intercepted and attenuated by the rarefaction wave before reaching the rear free surface. The shock wave after unloading retains some intensity, but the extent of fracture and phase transformation in the sphere is notably reduced. A comparison between Test 2 and Test 4 reveals that reducing the bumper thickness from 1 mm to 0.5 mm significantly shortens the rarefaction wave chasing distance, decreases the sphere’s fragmentation and phase transformation, and consequently increases damage to the rear wall.

### 4.2. Numerical Simulation Results and Validation

**Figure 16:**
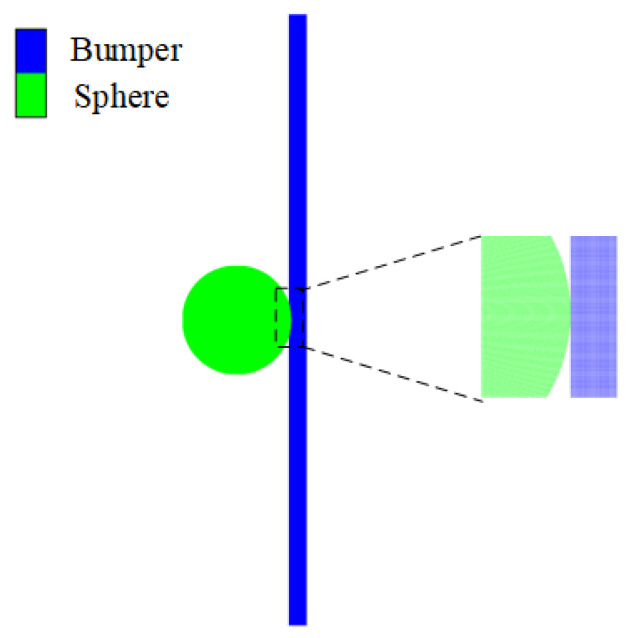
Simulation model of the sphere impacting the bumper.

To study the development process of the sphere impacting the bumper, smoothed particle hydrodynamics (SPH) simulations were conducted using AUTODYN 2023 R1. SPH is a robust method for replicating hypervelocity impact scenarios. The shock equation of state, the Johnson–Cook strength model, and the Grady spall failure model were utilized to describe the bumper material Al 5A06 [46]. For the polypropylene sphere, the polyethylene model from the AUTODYN material library was applied, as it provides a suitable approximation for the mechanical behavior of polypropylene under high-strain rate conditions. The 2D axial symmetry model was used to simplify the computational complexity while maintaining the essential features of the impact process, where the radius of the sphere was 3.5 mm and the bumper width was 20 mm (corresponding to 40 mm in the full-scale model). The bumper thickness and impact velocity were consistent with the experimental data in Table 1. Both the sphere and bumper SPH particles had a size of 0.02 mm, with a total of 47,934 particles in the sphere and 50,000 particles in a 1 mm thick bumper. Prior to impact, the sphere was in contact with the bumper, as depicted in Figure 16, which shows the initial configuration of the simulation.

In the above simulation model, the materials of the sphere and the bumper are considered to be ideal, homogeneous, and isotropic materials. These materials are assumed to have no internal defects, and their mechanical properties are uniform in all directions. The impact environment is an ideal vacuum. During the impact process of the sphere and the bumper, there is no heat exchange between the material and the environment. Additionally, the influence of the bumper boundary on the propagation characteristics of the internal shock wave is neglected.

**Figure 17:**
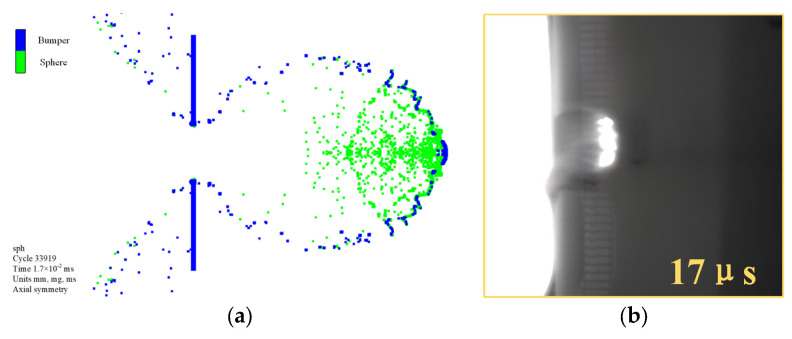
A comparison between simulation and experimental results of the debris cloud at an impact velocity of 3.03 km/s, 17 μs after impact. (**a**) Simulation result; (**b**) Experimental result.

Figure 17 compares the simulation and experimental results at an impact velocity of 3.03 km/s, demonstrating a close match between the debris cloud morphology from the simulation and the experimental observations at 17 μs after impact. Table 8 presents a comparison of the perforation sizes on the bumper, with errors between the simulation and experimental results all below 10%. This confirms that the simulation model accurately replicates the experimental results.

**Figure 18:**
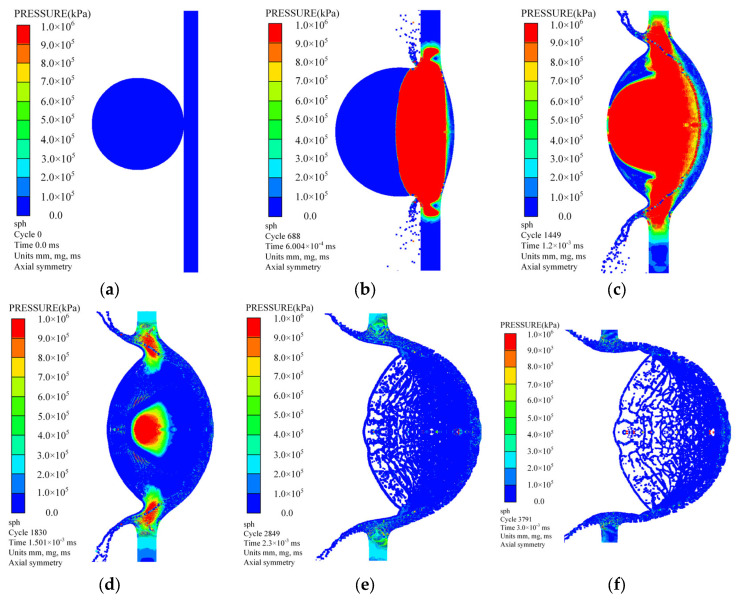
The complete process of perforation formation when a polypropylene sphere impacts the bumper at a velocity of 4.012 km/s. (**a**) At 0 μs; (**b**) At 0.6 μs; (**c**) At 1.2 μs; (**d**) At 1.5 μs; (**e**) At 2.3 μs; (**f**) At 3.0 μs.

Figure 18 illustrates the complete process of a polypropylene sphere impacting the bumper, resulting in a perforation at an impact velocity of 4.012 km/s. The perforation process can be delineated into three stages: In the initial stage, spanning from 0~1.2 μs post-impact, the sphere experiences compressive deformation along its axial direction and expansive deformation radially due to the shock wave, while the sphere also fractures. Concurrently, causing the bumper to deform and break under the combined impact of the sphere and its fragments, gradually forming a perforation. During this interaction, the internal energy of the sphere is swiftly transferred to the bumper. In the second stage, from 1.2~2.3 μs after impact, the shock wave propagates through the sphere and reaches the rear free surface, generating a rarefaction wave. This rarefaction wave travels within the sphere, gradually relieving the internal shock pressure. As the tensile force induced by the rarefaction wave increases, the spall initiates at the rear of the sphere. The axial dimension of the sphere elongates, while its radial expansion rate decreases. Consequently, the perforation size in the bumper increases with the radial expansion of the sphere and its fragments. The rate of energy transfer from the sphere to the bumper decelerates. In the final stage, beyond 2.3 μs, the internal pressure within the sphere is completely relieved. The radial expansion rate of the sphere ceases to increase, and the perforation size stabilizes after a slight increase due to inertial effects.

**Figure 19:**
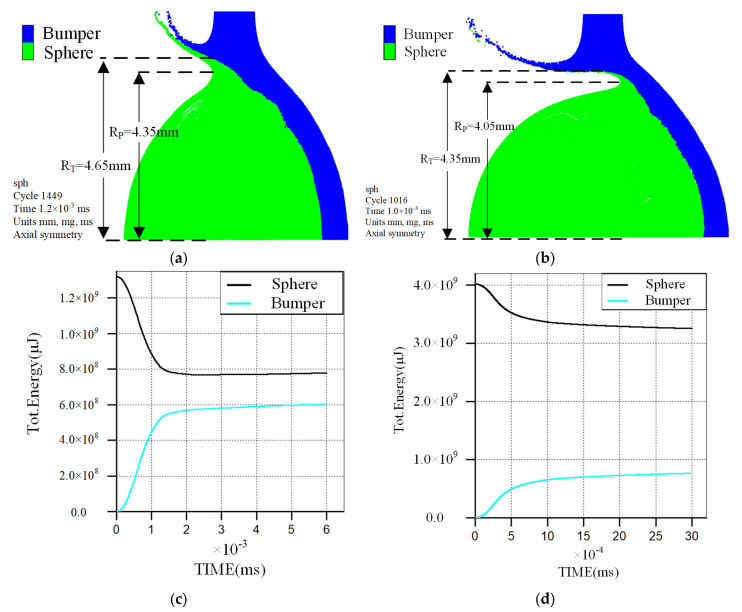
Deformation results and energy transfer curves of the sphere and bumper at an impact velocity of 4.012 km/s. (**a**) Deformation of polypropylene sphere and perforation size of the bumper; (**b**) Deformation of aluminum sphere and perforation size of the bumper; (**c**) Energy conversion process curve of polypropylene sphere and bumper; (**d**) Energy conversion process curve of aluminum sphere and bumper.

Figure 19 illustrates the deformation outcomes of polypropylene and aluminum spheres, as well as the energy transfer curves during perforation after the first stage of impact, with a velocity of 4.012 km/s. According to the material properties of aluminum, the impact pressure at this velocity is 41.63 GPa, and the shock wave velocity within the sphere is 8.016 km/s. From Figure 19a,b, it is evident that at the end of the first stage, the radial deformation radius of the polypropylene sphere is 4.35 mm, while that of the bumper impacted by the sphere and fragments is 4.65 mm. Conversely, the radial deformation radius of the aluminum sphere is 4.05 mm, and that of the bumper is 4.35 mm, both being smaller than those caused by polypropylene. Figure 19c,d show that during the impact of the polypropylene sphere with the bumper, the energy transfer time for the sphere’s kinetic energy is approximately 2.3 μs, with the bumper energy stabilizing at around 580 J and an energy transfer ratio of about 44%. For the aluminum sphere, the energy transfer time for its kinetic energy is about 1.0 μs, with the bumper energy stabilizing at approximately 630 J and an energy transfer ratio of about 15.7%.

Compared to aluminum, polypropylene exhibits lower density and yield strength, making it more susceptible to deformation and fragmentation. While the impact pressure and initial kinetic energy generated at the same velocity are smaller, polypropylene spheres undergo greater radial deformation and fragment more extensively under shock wave effects. This results in a larger perforation size on the bumper due to the sphere’s deformation and fragmentation. Moreover, the slower propagation speed of shock waves in polypropylene delays the formation of rarefaction waves and prolongs the recovery time for internal pressure to return to its initial state. During this recovery phase, particle velocity within the sphere continues to evolve with pressure, sustaining interaction with the bumper. This extends the duration of energy transfer from the sphere to the bumper, increasing the energy transfer ratio. Consequently, the polypropylene sphere retains significantly less residual energy post-impact than aluminum, despite imparting comparable total energy to the bumper. A higher energy transfer ratio from polypropylene impacts results in a lower velocity of the fragment cloud, reducing its damaging potential.

**Figure 20:**
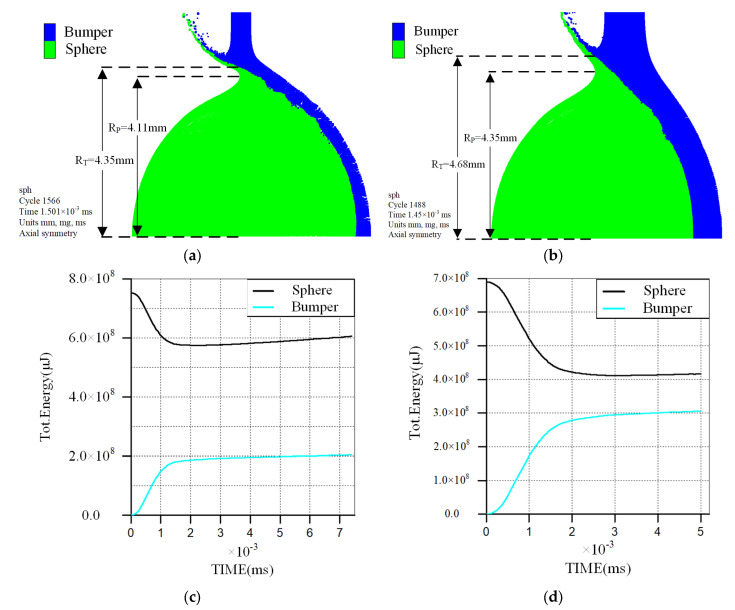
The results after the first stage of the polypropylene sphere impact under varying bumper thickness conditions. (**a**) Deformation of polypropylene sphere and perforation size of the bumper at an impact velocity of 3.03 km/s, bumper thickness of 0.5 mm; (**b**) Deformation of aluminum sphere and perforation size of the bumper at an impact velocity of 2.90 km/s, bumper thickness of 1.0 mm; (**c**) Energy conversion process curve of polypropylene sphere and bumper at an impact velocity of 3.03 km/s, bumper thickness of 0.5 mm; (**d**) Energy conversion process curve of aluminum sphere and bumper at an impact velocity of 2.90 km/s, bumper thickness of 1.0 mm.

Figure 20 presents the results after the first stage of the polypropylene sphere impact under varying bumper thickness conditions. At an impact velocity of 3.03 km/s and a bumper thickness of 0.5 mm, the deformed radial radius of the polypropylene sphere is 4.11 mm, while the radial radius of the bumper, subjected to the impact of the sphere and fragments, is 4.35 mm. The energy transfer time of the sphere’s kinetic energy is approximately 2.1 μs, the stable energy of the target plate is about 180 J, and the energy transfer ratio is roughly 24.6%. For an impact velocity of 2.9 km/s and a bumper thickness of 1 mm, the deformed radial radius of the polypropylene sphere increases to 4.35 mm, and the radial radius of the bumper under the impact of the sphere and fragments reaches 4.68 mm. The energy transfer time of the sphere’s kinetic energy extends to about 2.8 μs, the stable energy of the bumper rises to approximately 293 J, and the energy transfer ratio increases to 42.5%. Under similar impact velocities, increasing the bumper thickness results in greater radial deformation of the sphere, prolonged interaction time between the sphere and the bumper, and a higher energy transfer ratio. This leads to a reduction in the residual energy of the sphere and an increase in the bumper’s energy at stability, ultimately enlarging the perforation size of the bumper and diminishing the velocity of the debris cloud.

## 5. Conclusions

This study examines the damage characteristics of Whipple shields and the behavior of debris clouds under varying impact conditions by performing hypervelocity impact experiments with 7 mm diameter polypropylene spheres. Based on experimental results, combined with shock wave theory and numerical simulation analysis, the following conclusions can be drawn:

1. The polypropylene sphere transfers greater energy to the bumper upon impact, leading to a larger perforation size and reduced residual kinetic energy of the debris cloud. Compared to the aluminum sphere under the study’s impact conditions, the normalized diameter of bumper perforation caused by the aluminum sphere ranges from 1.13 to 1.41, whereas that caused by the polypropylene sphere ranges from 1.28 to 1.51, resulting in a significantly larger perforation size from the polypropylene sphere impacts.
2. The polypropylene sphere, with its low strength and phase transition susceptibility, produces a debris cloud primarily of ‘liquid filaments’ upon bumper impact. This cloud results in ‘groove’-shaped damage on the rear wall. Conversely, the aluminum sphere generates particle or droplet-like debris clouds, causing craters or perforations on the rear wall. The debris clouds and resultant damage morphology differ markedly between the two materials.
3. The Whipple shield damage characteristics and the debris cloud characteristics caused by the polypropylene sphere impacts are largely determined by impact velocity and the bumper thickness. The lower initial kinetic energy and higher energy dissipation caused by fracture and phase transitions reduce the sphere’s overall damage potential to the rear wall. Under the specified impact conditions, the aluminum sphere is capable of penetrating the rear wall, while the polypropylene sphere merely causes the ‘groove’ of varying sizes in the rear wall.

In future research work, the number of tests at different impact velocities and bumper thicknesses will continue to increase. Accurate empirical equations describing the characteristics of the debris cloud and the damage characteristics of the Whipple shield will be established. By combining numerical simulations, the relationship between impact conditions and the state of the debris cloud will be quantitatively analyzed.

## Figures and Tables

**Table 1:** Data of the 4 tests.

Test	Sphere Diameter (mm)	Impact Velocity (km/s)	Bumper Thickness (mm)	Rear Wall Thickness (mm)
1	7.0	2.257	1.0	2.0
2	7.0	2.900	1.0	2.0
3	7.0	4.012	1.0	2.0
4	7.0	3.030	0.5	2.0

**Table 2:** Diameter of bumper perforation from Test 1 to Test 4.

Test	Impact Velocity (km/s)	Bumper Thickness (mm)	Perforation Diameter (mm)	Normalized Diameter of Bumper Perforation
1	2.257	1.0	8.98	1.28
2	2.900	1.0	10.28	1.47
3	4.012	1.0	10.59	1.51
4	3.030	0.5	9.98	1.43

**Table 3:** Polypropylene debris cloud velocity from Test 1 to Test 4.

Test	Impact Velocity (km/s)	Bumper Thickness (mm)	Movement Velocity (km/s)	Expansion Velocity (km/s)
1	2.257	1.0	1.503	0.159
2	2.900	1.0	2.045	0.403
3	4.012	1.0	2.963	0.877
4	3.030	0.5	2.546	0.626

**Table 4:** Maximum average ‘groove’ depth from Test 1 to Test 4.

Test	Impact Velocity (km/s)	Bumper Thickness (mm)	Maximum Average ‘Groove’ Depth (mm)
1	2.257	1.0	0.35
2	2.900	1.0	0.19
3	4.012	1.0	0.16
4	3.030	0.5	0.26

**Table 5:** Material parameters of polypropylene and aluminum.

Material	Density (g/cm^3^)	Sound Speed (km/s)	Hugoniot Coefficient	Gruneisen Coefficient	Specific Heat (J/kg·K)	Thermal Expansion Coefficient (K^−1^)	Melting Temperature (K)
Polypropylene	0.874	4.093	1.253	0.87	1900	1.2 × 10^−4^	436
Al 5A06	2.640	5.328	1.340	2.0	850	0.714 × 10^−4^	864

**Table 6:** Pressure and temperature of polypropylene spheres under different impact conditions.

Test	Impact Velocity (km/s)	Shock Wave Speed of Polypropylene Spheres (km/s)	Shock Wave Pressure of Polypropylene Spheres (GPa)	Shock Temperature (K)	Residual Temperature (K)
1	2.257	6.236	9.32	584.2	452.6
2	2.900	6.813	12.93	787.4	578.5
3	4.012	7.792	20.11	1303.2	877.0
4	3.030	6.929	13.71	835.8	607.8

**Table 7:** The position where the axial rarefaction wave overtakes the shock wave under different impact conditions.

Test	Impact Velocity (km/s)	Bumper Thickness (mm)	Distance of the Chase Position from the Initial Impact Point (mm)
1	2.257	1.0	6.5417
2	2.900	1.0	6.3245
3	4.012	1.0	6.0138
4	3.030	0.5	3.0635

**Table 8:** Comparison of simulation and experimental results for bumper perforation dimensions.

Test	Impact Velocity (km/s)	Simulation Results (mm)	Experimental Results (mm)	Error
1	2.257	9.44	8.98	5.1%
2	2.900	10.38	10.28	1.0%
3	4.012	11.15	10.59	5.3%
4	3.030	9.83	9.89	−0.6%

## Data Availability

Data are contained within the article.

## Nomenclature

V_0_	impact velocity
V_1_	velocity of the front endpoint of the debris cloud
V_2_	velocity of the upper endpoint of the debris cloud
V_1_/V_0_	normalized velocity of the front endpoint of the debris cloud
V_2_/V_0_	normalized velocity of the upper endpoint of the debris cloud
C	sound speed of the bumper material
U	shock wave velocity
u	particle velocity
$c_{as}$	rarefaction wave speed
s	Hugoniot parameters
$x_{M}$	location where the axial rarefaction wave overtakes the shock wave
t	bumper thickness
S	distance between the bumper and the rear wall
D	diameter of the sphere
D_b_	diameter of the bumper perforation
D_b_/D	normalized diameter of bumper perforation
Dr	diameter of the rear wall damage area
ρ	density of the material
p	shock pressure
e	internal energy of the material
γ	Gruneisen coefficient
v	specific volume
T	temperature
$C_{v}$	specific heat
α	thermal expansion coefficient
